# Transcriptional Protein-Protein Cooperativity in POU/HMG/DNA Complexes Revealed by Normal Mode Analysis

**DOI:** 10.1155/2013/854710

**Published:** 2013-11-13

**Authors:** Debby D. Wang, Hong Yan

**Affiliations:** Department of Electronic Engineering, City University of Hong Kong, 83 Tat Chee Avenue, Kowloon, Hong Kong

## Abstract

Biomolecular cooperativity is of great scientific interest due to its role in biological processes. Two transcription factors (TFs), Oct-4 and Sox-2, are crucial in transcriptional regulation of embryonic stem cells. In this paper, we analyze how Oct-1 (a similar POU factor) and Sox-2, interact cooperatively at their enhancer binding sites in collective motions. Normal mode analysis (NMA) is implemented to study the collective motions of two complexes with each involving these TFs and an enhancer. The special structure of Oct proteins is analyzed comprehensively, after which each Oct/Sox group is reassembled into two protein pairs. We subsequently propose a segmentation idea to extract the most correlated segments in each pair, using correlations of motion magnitude curves. The median analysis on these correlation values shows the intimacy of subunit POUS (Oct-1) and Sox-2. Using those larger-than-median correlation values, we conduct statistical studies and propose several protein-protein cooperative modes (*S* and *D*) coupled with their subtypes. Additional filters are applied and similar results are obtained. A supplementary study on the rotation angle curves reaches an agreement with these modes. Overall, these proposed cooperative modes provide useful information for us to understand the complicated interaction mechanism in the POU/HMG/DNA complexes.

## 1. Introduction

Embryonic stem cells (ES cells) possess the pluripotency of differentiating into all the three germ layers (endoderm, mesoderm, and ectoderm), which correspond to hundreds of cell types. These pluripotent stem cells are transcriptionally regulated by a number of transcription factors (TFs) [1]. A specific TF called Oct-4, belonging to the POU class of homeodomain proteins, is regarded as a necessity for maintaining the undifferentiated state of embryonic ES cells. Generally, Oct-4 interacts with other TFs as a group to affect the gene expression of mouse ES cells in early embryo development [2], and Oct-4 coupled with its cofactor Sox-2 (HMG-box domain) is at the center of this group. Botquin and Nishimoto have both proven the cooperative effects of Oct-4 and Sox-2 on the expression of several genes in mouse embryonic ES cells [3, 4]. Dailey and Basilico further bring forward the idea that the interaction within the POU/HMG group, especially for groups composed of Oct and Sox proteins, at DNA binding sites is a fundamental mechanism for transcriptional regulation in early embryo development [5]. 

At the early stage of transcription, TFs bind to specific regulatory DNA regions to cooperatively affect the transcription sites. Enhancers, which act as activators or stimulators for transcription [6], are a major type of regulatory DNA regions. Unlike promoters, enhancers may be located kilobases away from their target genes, but geometrically they are most probably close to the genes due to the supercoil structure of DNA molecules, and thus there can be direct contacts between the enhancer-TF complexes and the transcription sites. Studies on the enhancer-TF complexes are very important for understanding the complicated mechanism of transcriptional regulation.

On the other hand, molecular dynamics are involved in many biological processes [7, 8], such as reproduction, regulation of gene expression, and protein interaction. As an indispensable component of gene expression, transcription must undergo a series of dynamical changes of biomolecules. Therefore, studies on dynamics of the aforementioned enhancer-TF complexes would provide a deep insight into their properties and functions in the transcriptional regulation. Specifically, deciphering the roles of Oct and Sox in the interaction mechanism of their enhancer-bounded complexes in the collective dynamics is of great scientific interest. Further, the cooperativity of the two proteins is a major research topic in these studies.

In our work, the dynamics of the POU/HMG group at its enhancer binding sites, referred to as POU/HMG/DNA complexes, are surveyed. Two POU/HMG/DNA complexes, which are DNA-binding portions of a POU factor Oct-1 and an HMG factor Sox-2 bound to an enhancer, are specifically studied from a structural and molecular dynamic view. Normal mode analysis (NMA) is implemented to study the collective or cooperative motions of these POU/HMG/DNA ternary complexes, after which the interaction of the POU and HMG factors at their DNA binding sites in these collective motions is explored. We propose a segmentation idea for the proteins to construct an equal-length-chain comparison and measure the correlation of each protein segment pair using the linear correlation. A statistical analysis on the significantly correlated pairs provides useful information on how these TFs have a synergistic control on enhancer DNAs in transcriptional regulation.

## 2. Materials and Method

### 2.1. Normal Mode Analysis (NMA)

#### 2.1.1. Introduction

NMA is an efficient method to detect the most cooperative or collective motions (essential modes) of large harmonic oscillating systems. With the constraint that the studied conformations are in the vicinity of the systematic equilibrium, which exists in most harmonic oscillating systems [9], NMA is useful for studying large structural deformations or motions of these systems. The idea is to use harmonic potentials to approximate a multidimensional energy landscape around an energy minimum for a system and to detect the most easily accessible modes on this energy landscape. NMA is broadly used to analyze the structural dynamics of biomolecules.

Specifically, if we describe an *N*-site-system with a position vector *q*, in which the three-dimensional coordinates of each site (*x*_1_, *y*_1_, *z*_1_), (*x*_2_, *y*_2_, *z*_2_),…, (*x*_*N*_, *y*_*N*_, *z*_*N*_) are used, we can mathematically expand the potential energy *V* in a second-order Taylor series around the equilibrium conformation *q* ^0^ [9]. Finally, we obtain a quadratic approximation as follows:

(1) $$\begin{array}{c} V\left(q\right)=\frac{1}{2}\sum_{i,j}{\left(\frac{\partial^{2}V}{\partial q_{i}\partial q_{j}}\right)}^{0}\left(q_{i}-q_{i}^{0}\right)\left(q_{j}-q_{j}^{0}\right)=\frac{1}{2}\Delta q^{T}H\Delta q. \end{array}$$

Here Δ*q* stands for the systematic structural changes relative to *q* ^0^, and *H* is a 3*N* × 3*N* Hessian matrix, whose elements have the following form:

(2) $$\begin{array}{c} H_{ij}=\frac{\partial^{2}V}{\partial q_{i}\partial q_{j}}. \end{array}$$

Subsequently, the kinetic energy is brought in to slightly modify the Hessian to a mass-weighted one. These Hessian matrices contain key structural information for our observed systems.

One broadly used construction method for the Hessian matrices is the elastic network models (ENMs) [9–12], which include the Gaussian network models (GNMs) [11] and anisotropic network models (ANMs) [12] as representatives. When ENM is applied, the equilibrium exploration can be skipped since the starting state is designed for this equilibrium. When constructing the ENM structure, the original system can be transformed into a network with nodes (CG-sites) and connecting springs, and a cutoff distance *γ*_*c*_ is used to define all the connecting springs *γ*_*ij*_ [9, 10]. Gaussian network model (GNM) selects representatives for substructures in the system, such as using C_*α*_-atoms for amino acids [9, 11], to further lower the computational cost, leading to the potential form shown as (3) (*R*_*i*_ or *R*_*j*_ represents a CG-site):

(3) $$\begin{array}{c} V_{\text{GNM}}=\frac{1}{2}\sum_{i,j}{\gamma_{ij}\left(\Delta R_{i}-\Delta R_{j}\right)}^{2}. \end{array}$$

Similarly, ANM proposes the potential form in (4) and ignores some influences caused by the distance vectors:

(4) $$\begin{array}{c} V_{\text{ANM}}=\frac{1}{2}\sum_{i,j}{\gamma_{ij}\left(||R_{i}^{0}+\Delta R_{i}-R_{j}^{0}-\Delta R_{j}||-||R_{i}^{0}-R_{j}^{0}||\right)}^{2}. \end{array}$$

Each eigenvalue of an above-constructed Hessian matrix denotes the associated systematic energy for the observed system, and its corresponding eigenvector represents the direction of a specific normal mode motion. Among the obtained 3*N* normal mode directions, the first six are trivial since they all correspond to zero eigenvalues, which means these structural changes have no effect on the systematic potential energy. For the remaining 3*N* − 6 eigenvectors, we will select a small set that corresponds to small eigenvalues (essential modes) for analysis [9]. In previous research, the first 10~15 essential modes are chosen by many researchers for their work [13–15], and the first 10 are analyzed in our work.

#### 2.1.2. Computational Platform

Several online tools are available for normal mode calculations. An online server called NOMAD-Ref at http://lorentz.immstr.pasteur.fr/nomad-ref.php [16] is utilized in our experiments. It is an ENM model-based method. The implementation of a rotation-translation block approach [16] and an ARPACK library for the sparse matrix data storage and decomposition [17] in the computations of Hessian matrices makes it possible to retain up to 100,000 atoms for each structure. In our work, when calculating the motions using NOMAD-Ref, all atoms in POU/HMG/DNA ternary complexes are used, while only motions of the POU and HMG proteins are analyzed since only protein-protein interactions in POU/HMG complexes at the DNA binding sites are of interest here.

### 2.2. Experimental Data and the Analysis on Their NMA Results

#### 2.2.1. Experimental Data

**Figure 1:**
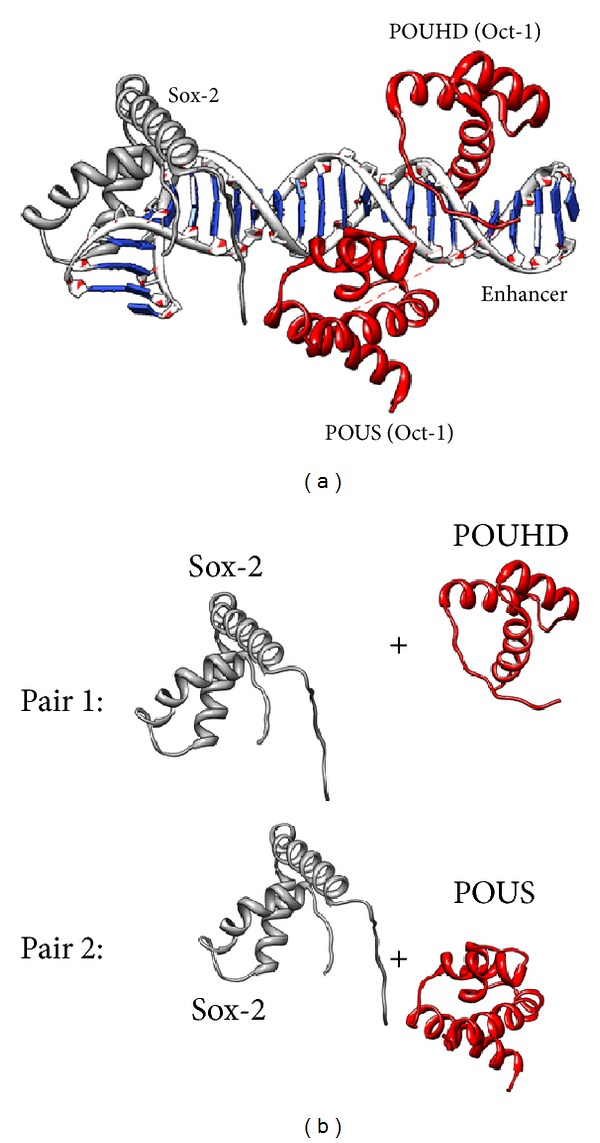
(a) The 3D structure of the POU/HMG/DNA ternary complex 1GT0. The gray protein represents an HMG factor Sox-2, and the red one is a POU factor Oct-1, which is composed of two subunits POUS and POUHD. (b) The two reassembled protein pairs, originated from (a), for our subsequent studies.

Two POU/HMG/DNA ternary complexes, 1GT0 and 1O4X, are downloaded from the Protein Data Bank (PDB) [18] for analysis. Each structure is composed of a POU factor Oct-1 (very similar to Oct-4), an HMG factor Sox-2 and an enhancer element.  Figure 1(a) displays the 3D structure of complex 1GT0 and the diagram is produced using UCSF Chimera [19]. In 1GT0, the bounded DNA piece is a fibroblast growth factor 4 enhancer (FGF4) [20]; in 1O4X, the homeobox B1 (Hoxb1) enhancer is bounded by the two TFs [21].

Furthermore, each Oct protein contains two subunits (POUS and POUHD) that are connected by a flexible linker and control DNAs in a bipartite manner [21]. Based on the special structure of Oct proteins, we regard Oct-1 and Sox-2 in each complex as the two protein pairs for further investigation, namely, POUHD and Sox-2 as pair 1 and POUS and Sox-2 as pair 2, both of which are shown in Figure 1(b).

#### 2.2.2. Analysis of Correlative Motions

After generating the motions of the two POU/HMG/DNA ternary complexes using NMA, we observe how the two protein pairs behave at the enhancer binding sites in these most collective or cooperative motions.

For each protein pair in each ternary complex, we analyze the first 10 obtained essential modes. In each mode, we firstly refine an observed pair at the residue level from a view of motion magnitude. This can be achieved by calculating the motion magnitudes for all the atoms in each protein and subsequently computing the motion magnitude of each residue in this protein by averaging the motion magnitudes of all component atoms (see (5)):

(5) MRi=1N∑j=1NiMAij=1N∑j=1Ni(xij−xij0)2+(yij−yij0)2+(zij−zij0)2.

**Figure 2:**
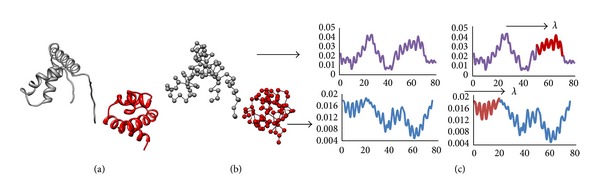
(a) The protein pair containing Sox-2 (gray) and POUS (red) in 1GT0. (b) The refined structure of the protein pair, where nodes represent residues. In each mode, a refined protein structure in the pair corresponds to a motion magnitude curve. (c) The searching process for the most cooperative segments of length *λ* in the protein pair in a specific mode.

Here atoms *j* = 1~*N*_*i*_ comprise the residue *i*; (*x*_*ij*_^0^, *y*_*ij*_^0^, *x*_*ij*_^0^) and (*x*_*ij*_, *y*_*ij*_, *x*_*ij*_) represent the positions of atom *j* in its equilibrium position and in a specific mode, respectively. Therefore, for each mode, we will obtain a motion magnitude curve for each protein in an observed pair, and each curve point corresponds to a residue along the protein sequence (Figures 2(a) and 2(b)).

Next, in each protein pair we observe the potential protein-protein cooperativity in these motions based on the correlations of motion magnitude functions. An effective method to measure the dependence between two quantities is the Pearson product-moment correlation coefficient [22–24] also is usually called the correlation coefficient. This coefficient is calculated based on the expected values (*μ*) and standard deviations (*σ*) of the two variables (**X** and **Y**), as shown in (6):

(6) $$\begin{array}{c} \text{corr}\left(X,Y\right)=\frac{\mathrm{cov}\left(X,Y\right)}{\sigma_{X}\sigma_{Y}}=\frac{E\left[\left(X-\mu_{X}\right)\left(Y-\mu_{Y}\right)\right]}{\sigma_{X}\sigma_{Y}}. \end{array}$$

We adopt this correlation coefficient in our studies. However, since each protein has a different length, we investigate the most cooperative/correlated segments among each protein pair in each mode. We introduce a segment length parameter *λ* here. For an observed pair of proteins that have different lengths of *x* and *y*, with a specific *λ* (*λ* ≤ *x* < *y*) defined in a mode, we shift one motion magnitude function along the other to find the *λ*-length-segments which share the largest absolute correlation value (Figure 2(c)). We could further describe the process as follows:

(7) max⁡ij C(i,j)=|corr(Segi,Segj)|So  that{Segi∈F1Segj∈F2||Segi||=||Segj||=λ.

Here *F*_1_ and *F*_2_ represent motion magnitude functions of the two proteins in an observed pair, in a specific essential mode; Seg_*i*_ and Seg_*j*_ denote *λ*-length-segments of *F*_1_ and *F*_2_, respectively.

In each modes with a list of *λ* values defined for each protein pair, we obtain a series of most cooperative segment pairs having correlation values *c*_*mn*_, where *m* denotes different *λ* values and *n* (1~10) represents different modes. Here we replace *λ* by *p* = *λ*/*x* for easier illustration and *x* is the shorter length in the observed pair. Since larger absolute value of correlation demonstrates more correlated segments (positively or negatively), we investigate how |*c*_*mn*_| distribute for the two protein pairs in each complex. For each *p* in an observed pair, the median value (8) is extracted and explored. Furthermore, the performances (based on ${\tilde{c}}_{m}$) of the two pairs in each complex are compared:

(8) c~m=MEDn=110{|cmn|}=MED{|cm1|,|cm2|,…,|cm10|},m=1,…,6.

Now, we use medians in (8) as a filter and investigate how those |*c*_*mn*_| larger than ${\tilde{c}}_{m}$ (supposed to be significant) distribute. For each protein in an observed pair, we can obtain a logic matrix *L* that reflects this process:

(9) L=[lmn]=([|cmn|]>[c~m]?)=([Cm]>[c~m]?)=((C1C2⋮C6)>(c~1c~2⋮c~6)?)So  that{Cm=(|cm1|,|cm2|,…,|cm10|)m=1,…,6n=1,…,10.

Here [*x*] gives the matrix that is composed of *x*. 

**Figure 3:**
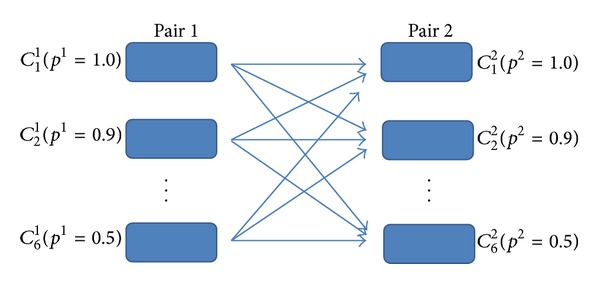
This diagram shows all the possibilities of the length pair (*p* ^1^, *p* ^2^). Specifically, for an observed complex, the significantly correlated segment pair (with a length parameter of *p* ^1^) in protein pair 1 and that (with a length parameter of *p* ^2^) in protein pair 2 are comparably investigated, with *p* ^1^ and *p* ^2^ taking all possible values (*m* = 1~6) as previously stated.

We subsequently examine the relationship between the two protein pairs in each complex based on these logic matrices. The idea is to explore that in a single essential mode whether only one significantly correlated segment pair (either in protein pair 1 or pair 2) is involved or both pairs are involved. To balance the segment lengths (*p*) used by the two pairs, we take into consideration all the length pairs (*p* ^1^, *p* ^2^) between the two pairs, as presented in Figure 3. Here we use superscripts to distinguish pairs 1 and 2. 

To fulfill the aforementioned operation, we conduct several iterations for all the *p* ^*i*^ values and combine the results of these iterations. We now take rows in *L* ^1^ (denoting a specific *p* ^1^ value) and show how the whole procedure is accomplished. In each iteration, we firstly expand the involved row (identified with a subscript *m*) into a matrix in (10) and then carry out statistics on the cases where three situations occur: (a) *S*_1_ (index *s*_1_)—only the significantly correlated segment pair in pair 1 is detected in a single essential mode with a length pair (*p* ^1^, *p* ^2^), (b) *S*_1_ (index *s*_2_)—only the significantly correlated segment pair in pair 2 is detected, and (c) *D* (index *d*)—both pairs 1 and 2 are detected. The statistical analysis is based on logic operations, as shown in (11), which combines all the iterations to derive the final indexes for the two pairs:

(10) L1,m=(Lm1,Lm1,…,Lm1︸  )T6,

(11) s1=∑m=16sum(L1,m·×(¬L2)),s2=∑m=16sum((¬L1,m)·×L2),d=∑m=16sum(L1,m·×L2).

Here “·×” means a batch of multiplications of the corresponding elements in two matrices, and sum(*X*) counts the number of ones (a logic “*true*” value) in a logic matrix *X*. Indexes *s*_1_, *s*_2_, and *d* separately show three cooperative modes (corresponding to the aforementioned three cases) between the two protein pairs in a POU/HMG/DNA complex. We exhibit some representative cooperative modes in Section 3, where we also list the above-mentioned indexes for the two complexes. Furthermore, to take the signs of the correlations *c*_*mn*_ into consideration, we introduce another logic matrix *Z* that describes the signs of *c*_*mn*_, as stated in (12). Through combining logic operations of *L* ^*i*^ and *Z* ^*i*^ (13), we can divide the situations (*S*_1_, *S*_2_, and *D*) into subtypes (positive and negative), and all these subtypes are analyzed in Section 3: 



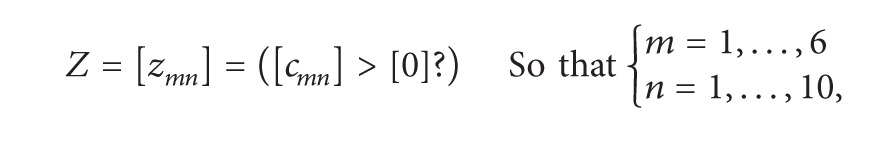
(12)

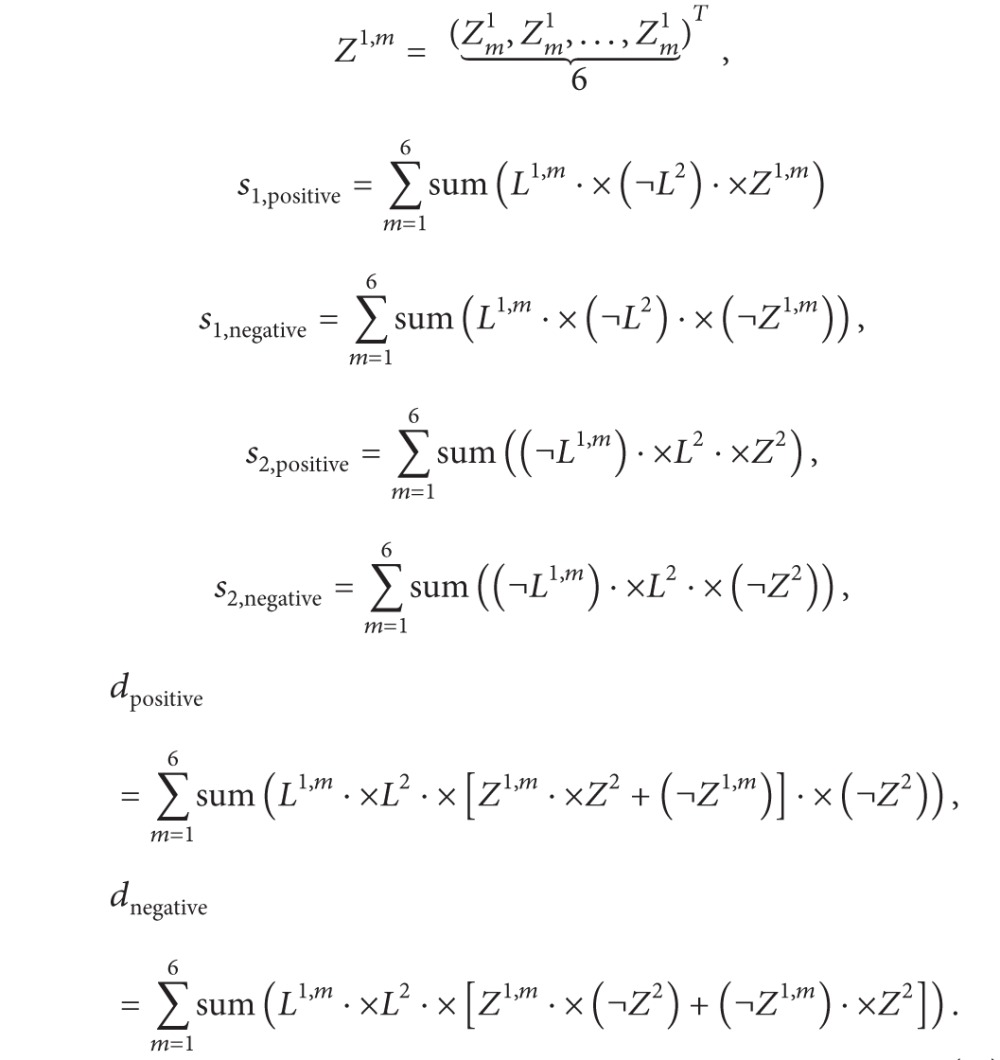
(13)


To compare the scenarios where different filters are applied, we, respectively, apply the first tertile, the first quartile, and the mean value as filters to investigate the corresponding results. The mean filter can be described as (14), and the quantile filter as (15), where Pr represents probability. Specifically, the tertile and quartile filters correspond to situations where *p* = 1/3 and *p* = 1/4, respectively. A series of operations are then carried out based on these filters, to reveal how the observed complexes behave in these situations:

(14) $$\begin{array}{c} {\overset{-}{c}}_{m}=\frac{\sum_{n=1}^{10}\left\{\left|c_{mn}\right|\right\}}{10},\;m=1,\ldots ,6, \end{array}$$

(15) c~m(p)=inf⁡⁡{|cmn| ∣ F(|cmn|)≥p},F(|cmn|)=Pr⁡(Cm≤|cmn|),m=1,…,6.

Finally, to gain a deep insight into the motions of these two complexes, we have also observed the rotation angles of the corresponding protein chains. In the above discussion, we regard residues as basic units in protein sequences, and here we consider the links between each consecutive two residues (Figure 2(b)). The angles between each pair of corresponding links in the original structure and in deformed structures (modes) are studied. We obtain a rotation angle function for each protein of a protein pair in each essential mode. Afterwards, we conduct a similar analysis as aforementioned on these rotation angle functions as a supplementary study. Principal component analysis (PCA) is implemented to reduce the effect of noisy rotation angles. We also investigate the suitability of the Fourier transform for data analysis.

## 3. Results and Discussion

### 3.1. Motion Magnitude Functions

**Figure 4:**
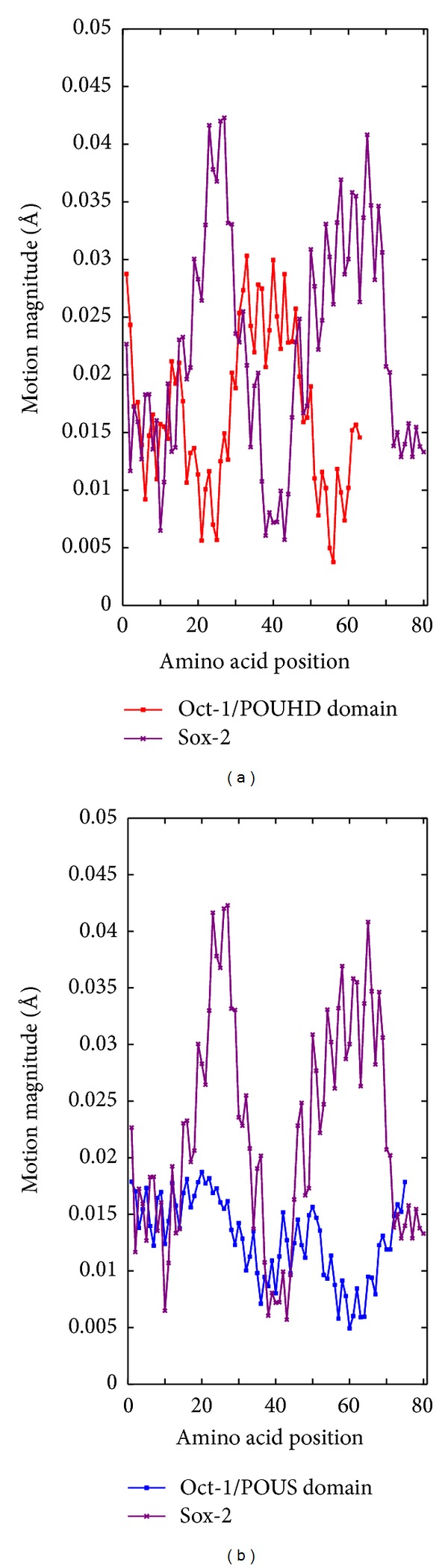
(a) The motion magnitude curves in mode 7 for proteins POUHD (red) and Sox-2 (purple) in pair 1 of 1GT0. (b) The motion magnitude curves in mode 7 for proteins POUS (blue) and Sox-2 (purple) in pair 2 of 1GT0.

For each protein in an observed pair of a ternary complex, we calculate the motion magnitude functions (5) for the first 10 essential modes.  Figure 4 shows the motion magnitude curves for the two observed protein pairs in 1GT0 for the first essential mode.

After defining a list of *p* values, we calculate the most cooperative/correlated segment pairs among each protein pair in a complex for the 10 essential modes, using the mechanism discussed in Section 2.2.2. Since small *p* values correspond to shorter segment matching, whose results may be trivial due to the high correlation possibilities, we use a set of *p* values starting at 0.5 to 1.0 at a step of 0.1.  Table 1 shows the results of correlations *c*_*mn*_ for the most cooperative segment pairs among protein pair 1 of 1GT0.

**Figure 5:**
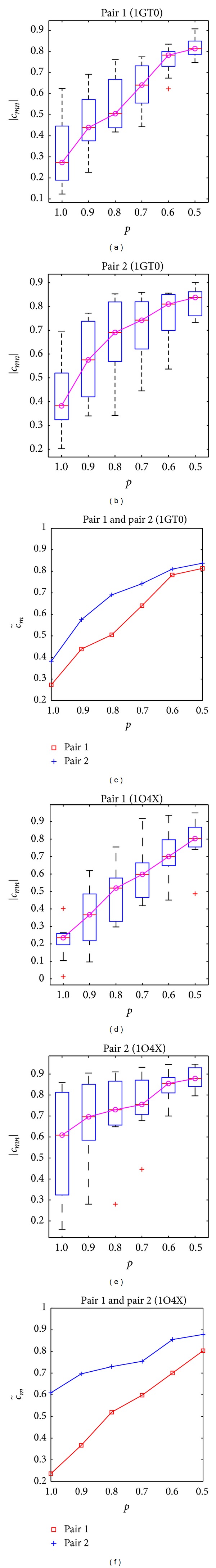
(a) and (b) show the distributions of absolute correlation values |*c*_*mn*_| for the two protein pairs in complex 1GT0, respectively. In (c), the median absolute correlation value ${\tilde{c}}_{m}$ for each *p* is extracted for the two pairs from (a) and (b). Similarly, (d), (e), and (f) are the plots for complex 1O4X.

The larger the absolute value of correlation is, the more the two compared segments correlate with each other, either positively or negatively. Now we examine how the absolute correlation values |*c*_*mn*_| distribute, for the two protein pairs in each complex. The values are presented in Figure 5, where parts (a) and (b), respectively, show the values for the two pairs in 1GT0, and parts (d) and (e) show those for 1O4X. We can see from these diagrams that |*c*_*mn*_| becomes larger when *p* gets smaller, and this can also be detected from the median value ${\tilde{c}}_{m}$ shown with a pink circle in each box (denoting a specific *p*). To give a comparison between the performances of the two pairs in each complex, we extract the above-mentioned median values ${\tilde{c}}_{m}$ for each pair and present them in parts (c) (1GT0) and (f) (1O4X). In diagrams (c) and (f), especially (f), pair 2 presents a higher ${\tilde{c}}_{m}$ than pair 1, which to some extent implies that pair 2 may behave as a leading role in the Oct/Sox interactions.

**Figure 6:**
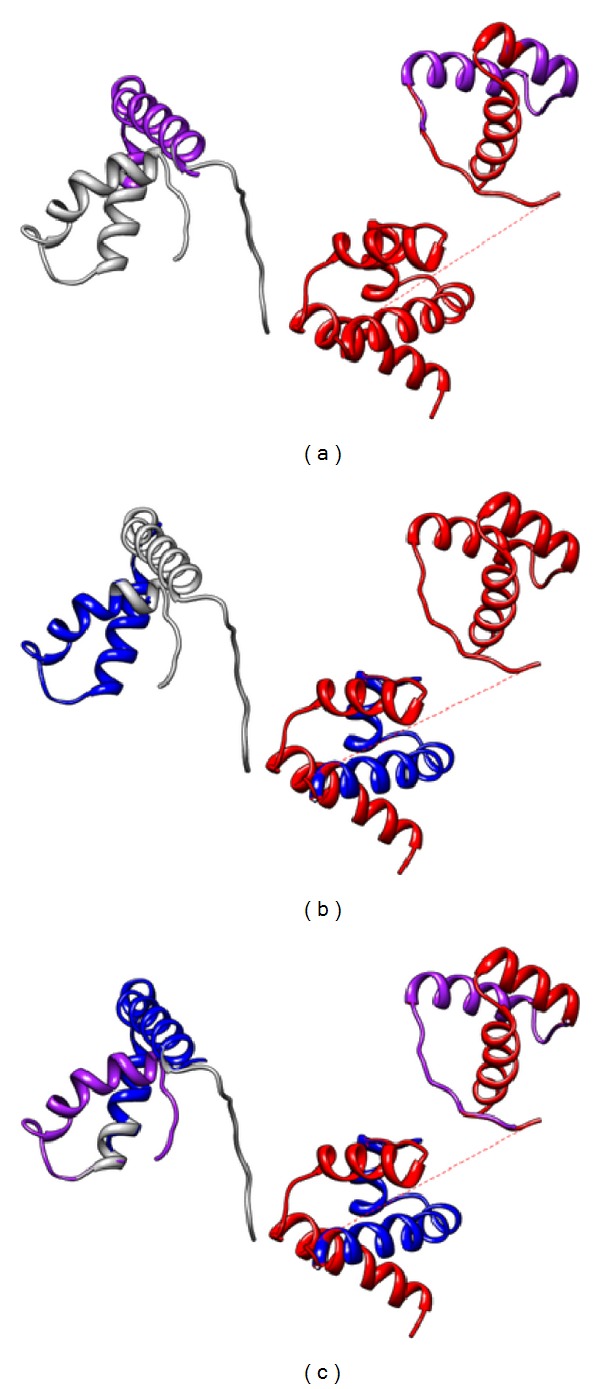
Parts of the results of cooperative modes *S* and *D* in complex 1GT0, with TFs Oct-1 and Sox-2 colored red and gray, for *p* ^1^ = *p* ^2^ = 0.5 in the first 10 essential modes. (a) displays mode *S*_1_ in normal mode 13, with the significantly correlated segments in protein pair 1 colored purple; (b) displays mode *S*_2_ in normal mode 12 and the correlated segments are colored blue in protein pair 2; (c) presents mode *D* in normal mode 16 with the correlated segment pairs colored purple and blue, respectively, in both protein pairs.

Next, we use the above-mentioned medians as a filter and investigate how those |*c*_*mn*_| larger than ${\tilde{c}}_{m}$ (supposed to be significant) distribute. For each protein in an observed pair, we calculate its logic matrices *L* and *Z* (Section 2.2.2), which correspond to (9) and (12), respectively. We subsequently study the logic matrices of the two protein pairs (*L* ^1^ and *Z* ^1^, *L* ^2^ and *Z* ^2^) in each complex, after which we propose several cooperative modes between the two pairs and conduct statistical analysis according to (10) and (11). In detail, these modes include (a) mode *S*_1_ (index *s*_1_)—only the significantly correlated segment pair in pair 1 is detected in a single essential mode with a length pair (*p* ^1^, *p* ^2^), (b) mode *S*_1_ (index *s*_2_)—only the significantly correlated segment pair in pair 2 is detected, and (c) mode *D* (index *d*)—both pairs 1 and 2 are detected. To visually show the cooperative modes *S* and *D*, we select parts of the results of 1GT0 for *p* ^1^ = *p* ^2^ = 0.5 as a display in Figure 6, in which *S*_1_, *S*_2_, and *D* modes are, respectively, presented with the significantly correlated segment pairs colored. 

Mode *S* denotes that only one protein pair, either pair 1 (*S*_1_) or pair 2 (*S*_2_), is significantly involved in a specific collective motion. This indicates that only one subunit, either POUHD or POUS, is significantly involved in the cooperativity with Sox-2 in an essential mode. Mode *D* implies that both subunits are involved in the interactions with Sox-2. Detailed statistical results are reported in Table 2. In this table, cooperative mode *D* occurs more frequently than modes *S*_1_ and *S*_2_ in the two complexes, which have the tuples of (82, 82, 98) and (85, 85, 95) for the indexes (*s*_1_, *s*_2_, *d*), respectively. This implies that, compared with mode *S*_1_ or *S*_2_, both subunits of Oct-1 frequently participate in the interactions with Sox-2 at the same time, as mode *D*. 

Furthermore, we divide the modes *S* and *D* into subtypes, positive subtype and negative subtype, and their statistics are evaluated using (13) and listed in Table 2. In modes *S*_1_ and *S*_2_, the positive subtype (*s*_1,positive_ and *s*_2,positive_) shows a positive sign of *c*_*mn*_ for the significantly correlated segment pair in protein pair 1 or 2, and the negative one (*s*_1,negative_ and *s*_2,negative_) indicates a negative sign. In mode *D*, the positive subtype (*d*_positive_) denotes a scenario where both significantly correlated segment pairs in the two protein pairs share the same sign of *c*_*mn*_ (+/+ or −/−), and the negative one (*d*_negative_) represents two different signs (+/− or −/+). From Table 2 we notice that, for mode *S*_2_ in both complexes 1GT0 and 1O4X, the positive subtype has a lead; for modes *S*_1_ and *D*, the negative subtype is in the lead for 1GT0 while the positive one is in a dominant position for 1O4X. 

We have also applied the first tertile, the first quartile and the mean value as filters and similarly conducted the statistical analysis as illustrated above. Tables 3, 4, and 5 present the results for these three scenarios, respectively. As shown in these tables, the gap between the occurrence frequencies of mode *D* and mode *S*_1_ (or *S*_2_) becomes larger, and the dominant occurrences of mode *D* are demonstrated. Besides, for modes *S*_1_ and *D*, complexes 1GT0 and 1O4X have the opposite subtype distributions, while for mode *S*_2_, they present a similar distribution. Overall, these additional results are consistent with the previous one (the median filter).

### 3.2. Rotation Angle Functions

**Figure 7:**
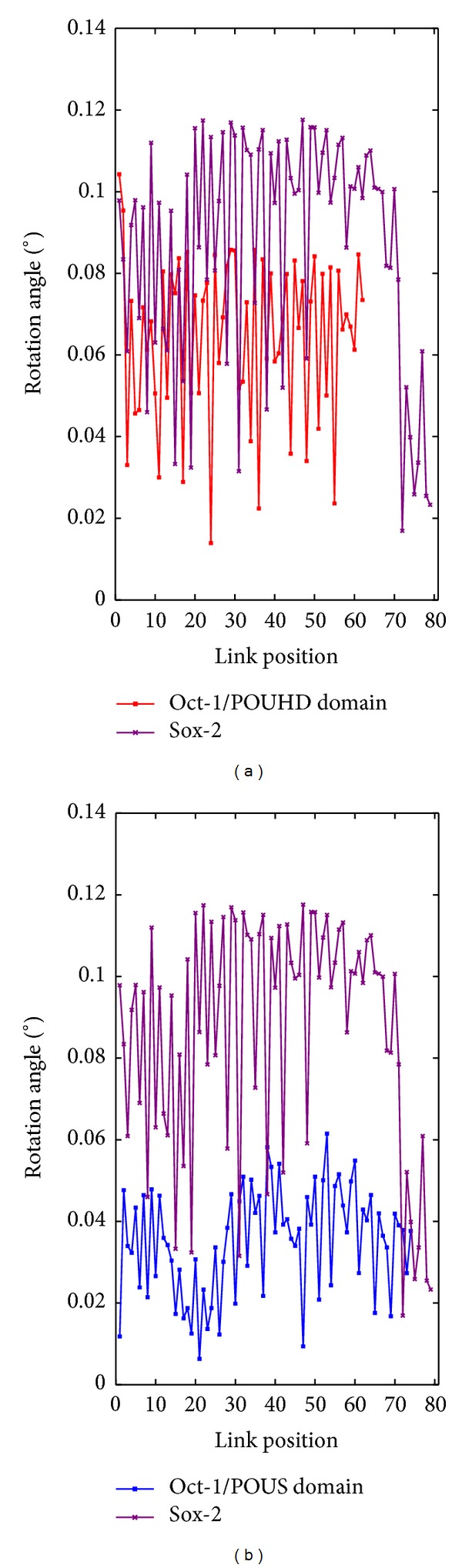
(a) displays the rotation angle curves for proteins POUHD (red) and Sox-2 (purple) in pair 1 of 1GT0 in mode 7; (b) shows the rotation angle curves for proteins POUS (blue) and Sox-2 (purple) in pair 2 of 1GT0 in mode 7.

Subsequently, we calculate the rotation angle functions for each protein in each complex in the first 10 essential normal modes (described in Section 2.2.2).  Figure 7 shows the rotation angle curves of proteins in the two protein pairs of 1GT0 in the first essential mode.

Since the rotation angle functions contain a lot of noise, we apply the principal component analysis (PCA) to the 10 rotation angle curves of each protein in the two complexes to obtain the first principal component (PC), leading the rotation angle curves (*n* = 1~10) of each protein to a single condensed PC curve. We similarly carry out the correlation analysis of the PC curves in each pair.  Table 6 shows the statistical results for the two complexes. Intuitively, 1GTO presents the distinct cooperative mode of *S*_1_, where pair 1 shows more significantly correlated segment pairs (with a positive subtype), while mode *D* is the dominant one in 1O4X, where many significantly correlated segment pairs occur in both pairs (with a positive subtype). 

Now we apply the Fourier transform to analyze these noisy rotation angle values. Simply, the magnitudes of the transformed signals are regarded as our new data. The segmentation and correlation calculation are implemented, after which the statistical analysis is carried out. As an example, we use the first quartile as a filter for the correlations of rotation angle functions. The results are listed in Table 7, where we can see that the negative subtype of each cooperative mode is concealed after the transform. This implies that the Fourier transform may not be a suitable tool for handling these rotation angle values. More efficient strategies should be explored in the future to deal with these data.

## 4. Conclusions

In this paper, we performed NMA to study the collective motions of two TFs, Oct-1 and Sox-2, at their enhancer binding sites, aiming to gain an insight into the cooperative manner of these two TFs through the dynamics of their enhancer-bounded complexes. Based on the special structure of Oct proteins, we treated an Oct/Sox group as two protein pairs and comparably investigated how these two pairs behave in the collective motions. A segmentation idea was introduced to explore the most correlated segments in each protein pair, according to the correlations of motion magnitude curves (or their segments). A median analysis on these correlations was conducted, which shows the leading role of subunit POUS (pair 2). Furthermore, based on statistics of the correlated segment pairs having a correlation value above the corresponding median, we proposed several motion cooperative modes (*S*_1_, *S*_2_, and *D*) and their subtypes (positive or negative). The first tertile, the first quartile, and the mean value provide consistent results. Moreover, the supplementary study on the rotation angle functions presents a consensus about these modes. These proposed modes provide a clue that when binding to different regulatory DNA regions or involved in different collective motions, Oct-1 has a synergistic relationship with Sox-2 either with one of the components, POUS or POUHD, or both of them, POUS and POUHD at the same time.

Cooperativity, in protein-DNA [25] and protein-protein [26] interactions, is an important feature in biomolecular interactions. In our work, we carried out a series of studies on the cooperative manner of Oct and Sox at their enhancer binding sites, which are important elements in the transcriptional regulation of embryonic stem cells. This work reveals how the two proteins work together physically and structurally at two specific DNA biding sites. The method developed here can be useful for the analysis of molecular interactions in other protein-protein and protein-DNA complexes.

## Figures and Tables

**Table 1:** Motion correlations between POUHD and Sox-2 in protein pair 1 of 1GT0.

*p*	Mode									
	Mode 7	Mode 8	Mode 9	Mode 10	Mode 11	Mode 12	Mode 13	Mode 14	Mode 15	Mode 16
1	−0.696	0.606	−0.477	0.324	−0.265	0.383	0.202	−0.326	−0.382	−0.520
0.9	−0.738	0.711	0.697	0.340	−0.419	0.772	0.454	−0.429	−0.420	0.739
0.8	−0.853	0.797	0.819	0.342	0.651	0.838	−0.607	−0.463	0.569	0.730
0.7	−0.859	0.836	0.820	−0.445	0.639	0.820	−0.621	−0.562	−0.716	0.769
0.6	−0.856	0.851	0.850	0.537	0.814	0.849	−0.699	−0.684	0.762	0.806
0.5	−0.865	−0.862	0.856	−0.757	0.858	−0.901	−0.733	−0.761	0.806	0.819

**Table 2:** Statistics on the occurrences of the cooperative modes *S* and *D*, and their subtypes, in the 10 essential modes for all the pairs (*p* ^1^, *p* ^2^), using the median filter for motion correlations.

1GT0								
*s*_1_			*s*_2_			*d*		
82	*s*_1, positive_	*s*_1, negative_	82	*s*_2, positive_	*s*_2, negative_	98	*d*_positive_	*d*_negative_
	36	46		49	33		35	63

1O4X								
*s*_1_			*s*_2_			*d*		
85	*s*_1, positive_	*s*_1, negative_	85	*s*_2, positive_	*s*_2, negative_	95	*d*_positive_	*d*_negative_
	62	23		75	10		68	27

**Table 3:** Statistics on the occurrences of the cooperative modes *S* and *D*, and their subtypes, in the 10 essential modes for all the pairs (*p* ^1^, *p* ^2^), using the first tertile as a filter for motion correlations.

1GT0								
s_1_			s_2_			*d*		
71	*s*_1, positive_	*s*_1, negative_	71	*s*_2, positive_	*s*_2, negative_	145	*d*_positive_	*d*_negative_
	28	43		47	24		70	75

1O4X								
s_1_			s_2_			*d*		
77	*s*_1, positive_	*s*_1, negative_	71	*s*_2, positive_	*s*_2, negative_	139	*d*_positive_	*d*_negative_
	57	20		62	9		98	41

**Table 4:** Statistics on the occurrences of the cooperative modes *S* and *D*, and their subtypes, in the 10 essential modes for all the pairs (*p* ^1^, *p* ^2^), using the first quartile as a filter for motion correlations.

1GT0								
*s*_1_			*s*_2_			*d*		
62	*s*_1, positive_	*s*_1, negative_	62	*s*_2, positive_	*s*_2, negative_	190	*d*_positive_	*d*_negative_
	26	36		33	29		85	105

1O4X								
*s*_1_			*s*_2_			*d*		
70	*s*_1, positive_	*s*_1, negative_	64	*s*_2, positive_	*s*_2, negative_	182	*d*_positive_	*d*_negative_
	47	23		52	12		119	63

**Table 5:** Statistics on the occurrences of the cooperative modes *S* and *D*, and their subtypes, in the 10 essential modes for all the pairs (*p* ^1^, *p* ^2^), using the mean value as a filter for motion correlations.

1GT0								
*s*_1_			*s*_2_			*d*		
66	*s*_1, positive_	*s*_1, negative_	78	*s*_2, positive_	*s*_2, negative_	108	*d*_positive_	*d*_negative_
	33	33		43	35		44	64

1O4X								
*s*_1_			*s*_2_			*d*		
73	*s*_1, positive_	*s*_1, negative_	85	*s*_2, positive_	*s*_2, negative_	107	*d*_positive_	*d*_negative_
	52	21		72	13		77	30

**Table 6:** Correlations between PC curves of rotation angle functions for the two protein pairs in 1GT0 and 1O4X.

*p*	1GT0		1O4X	
	Pair 1	Pair 2	Pair 1	Pair 2
1.0	0.682	−0.278	−0.875	−0.310
0.9	0.836	−0.339	−0.884	−0.764
0.8	0.844	−0.354	−0.884	−0.821
0.7	0.854	−0.328	−0.884	−0.829
0.6	0.863	−0.498	−0.890	−0.856
0.5	0.876	0.703	−0.915	−0.859

**Table 7:** Statistics on the occurrences of the cooperative modes *S* and *D*, and their subtypes, in the 10 essential modes for all the pairs (*p* ^1^, *p* ^2^), using the first quartile as a filter for the correlations of rotation angle functions.

1GT0								
*s*_1_			*s*_2_			*d*		
108	*s*_1, positive_	*s*_1, negative_	108	*s*_2, positive_	*s*_2, negative_	144	*d*_positive_	*d*_negative_
	108	0		108	0		144	0

1O4X								
*s*_1_			*s*_2_			*d*		
48	*s*_1, positive_	*s*_1, negative_	48	*s*_2, positive_	*s*_2, negative_	204	*d*_positive_	*d*_negative_
	48	0		48	0		204	0
